# Supplementary material for: Deep brain stimulation of thalamic nucleus reuniens promotes neuronal and cognitive resilience in an Alzheimer’s disease mouse model
Source: Nat Commun. 2023 Nov 2;14:7002. doi: 10.1038/s41467-023-42721-5 (PMC10622498; doi:10.1038/s41467-023-42721-5)
Supplement: Supplementary file 1 — Supplementary Information [file 41467_2023_42721_MOESM1_ESM.pdf]

Supplementary Information

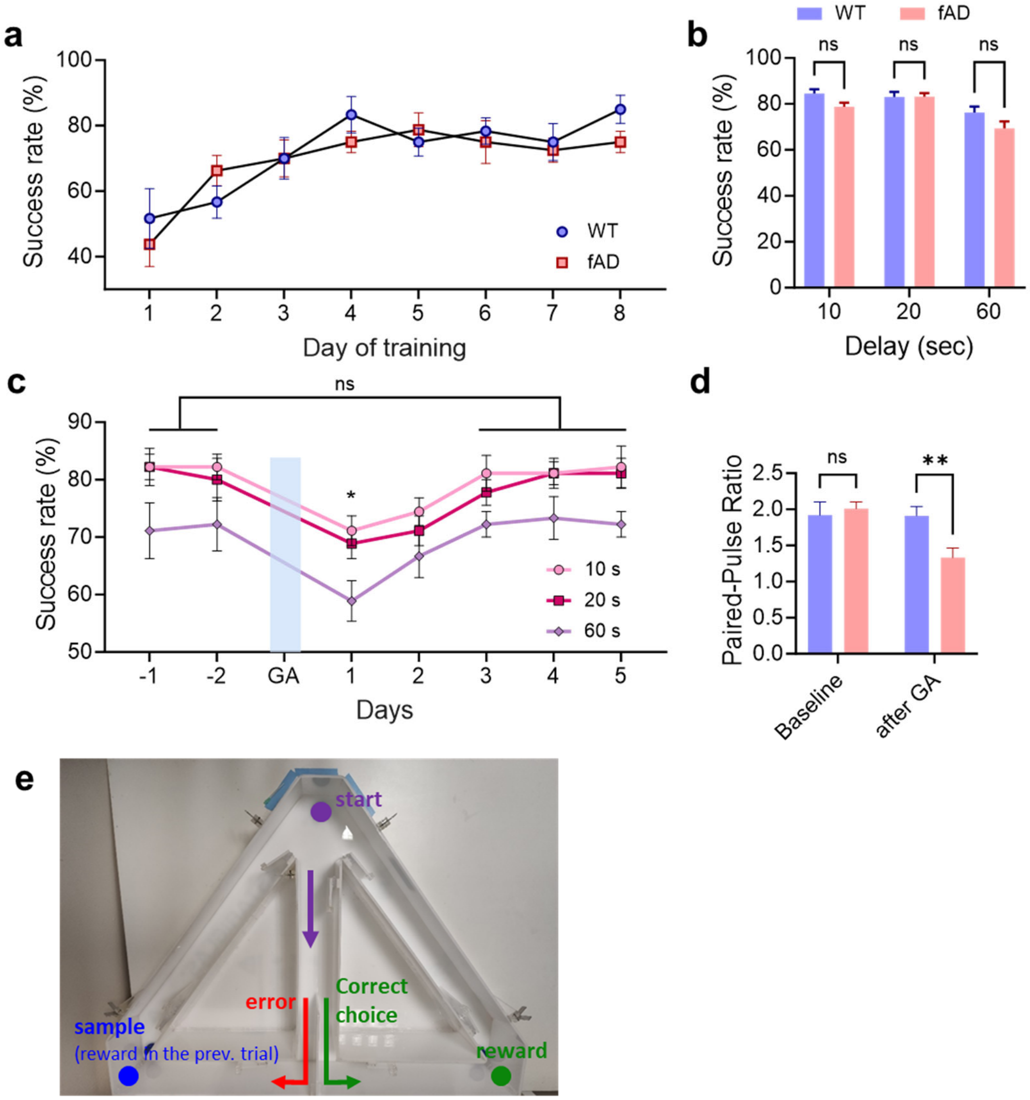

Figure S1 | Spatial working memory of 4-5-month-old fAD mice was not different than WT littermates. a Learning curve of spatial working memory (SWM) assessed by delta-maze was similar between genotypes (*P* = 0.64; Two-way ANOVA, WT n = 6, APP/PS1 n = 8). **b** No difference in SWM in delta-maze between 4-5 m.o. WT (n = 9) and fAD (n = 9) mice (Two-way ANOVA with Sidak's multiple comparison tests: 10 sec *P* = 0.329, 20 sec *P* > 0.999, 60 sec *P* = 0.1986). **c** Delta maze success rate of fAD mice before and after general anesthesia (GA). The impairment in SWM performance of fAD mice following GA was transient. **d** Paired-pulse ratio in the nRE-CA1 synapse was similar in WT (blue, n = 6) and fAD (red, n = 8) during 3 days of baseline, but lower in fAD in the 3 days following GA (*P* = 0.004, mixed effect analysis with Sidak's multiple comparison tests: baseline *P* = 0.8723, after GA *P* = 0.0078). **e** SWM was examined

using Delta-maze, a continuous variation of the T-maze. ns – non-significant, $**P < 0.01$. Error bars represent SEM.

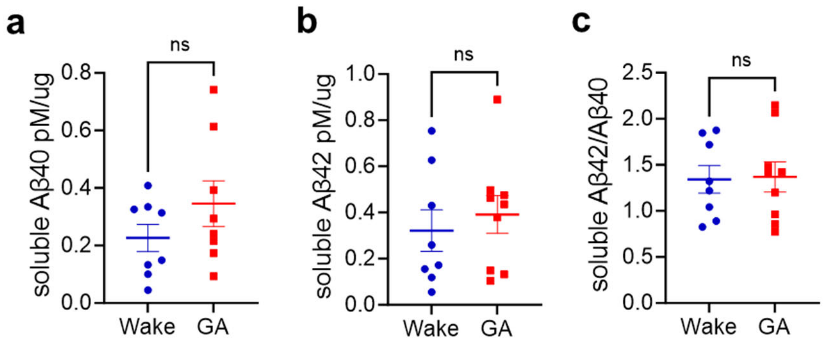

Figure S2 | **Soluble Aβ40 or Aβ42 levels in the hippocampus of APP/PS1 mice. a-b** Soluble Aβ40 (a) and Aβ42 (b) levels in awake (n = 8) and following GA (3hr, 1.5% isoflurane, n = 9) 5-m.o. APP/PS1 mice. *P* = 0.44 for Aβ40 and *P* = 0.61 for Aβ42. **c** Aβ42/Aβ40 ratio in soluble fraction (*P* = 0.96, the same data as in a,b). Mann-Whitney two-tailed u test was used for the analysis (a-c). ns – non-significant. Error bars represent SEM.

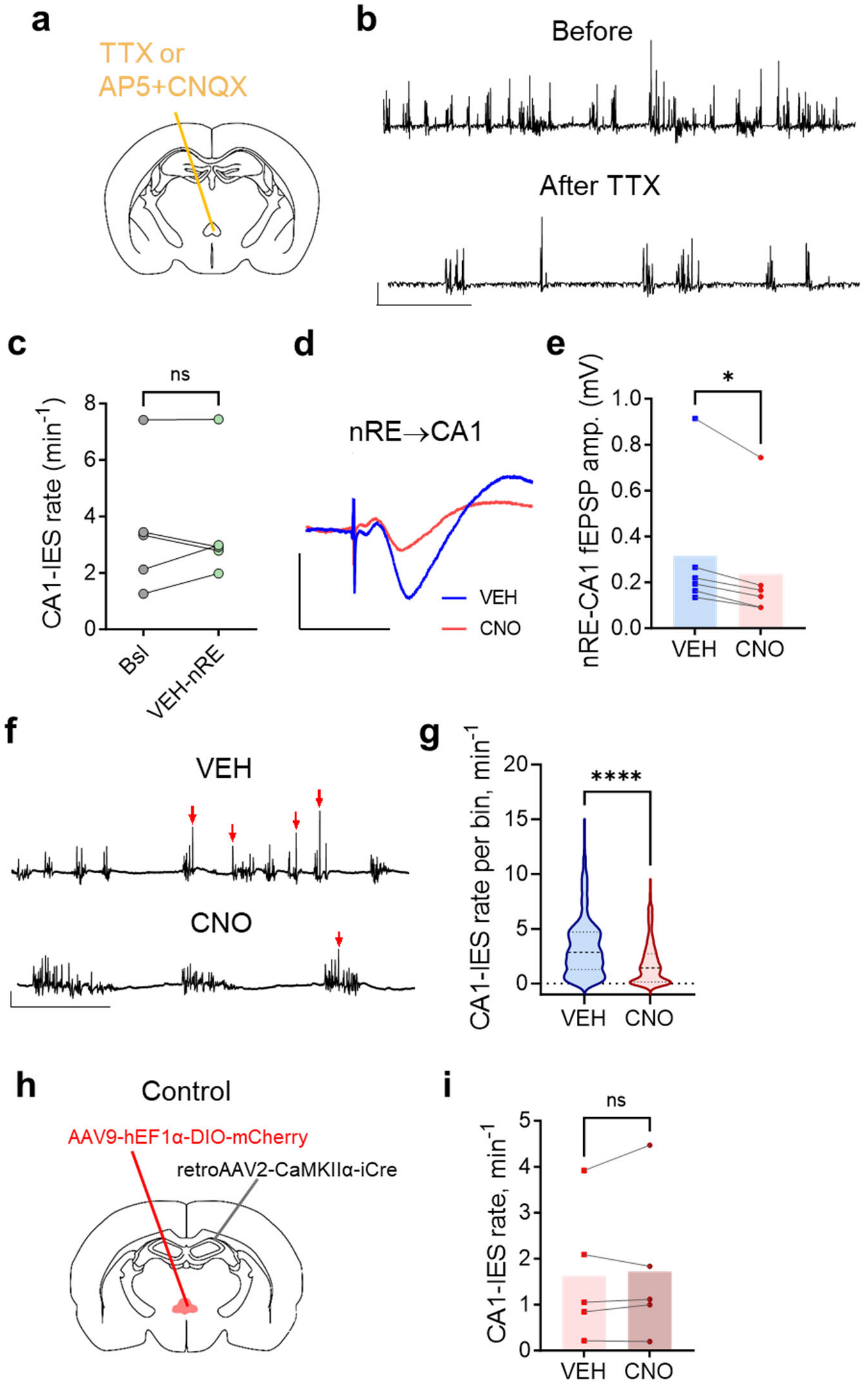

Figure S3 | **Pharmacological and pharmacogenetic inactivation of nRE. a** Illustration of local TTX / CNQX+ AP5 injection to the nRE. **b** Representative traces of CA1 LFP recordings, depicting interictal epileptiform spikes (IES) before and after local TTX injection to the nRE of anesthetized fAD mice. Scale bars: 0.5 mV, 20 sec. **c** Local injection of VEH (DMSO) to the nRE didn't cause a significant change in the mean rate of IES in anesthetized fAD mice during the 10-40 minutes following the injection, compared to the 30 minutes prior to injection (*P* = 0.6250, Wilcoxon two-

tailed test, n = 5). **d** Representative fEPSP recordings evoked by 0.15 mA stimuli at 0.06 Hz in nRE-CA1 synapse 30 min after VEH (saline) vs CNO i.p. injections (5 mg/kg). Scale bars: 0.5 mV, 20 ms **e** Effect of CNO versus VEH on the peak amplitude of fEPSP in the nRE-CA1 synapses of awake, head fixed APP/PS1 mice expressing hM4D(Gi)-mCherry in the nRE-CA1 synapses (*P* = 0.031, Wilcoxon two-tailed test, n = 6). **f** Representative traces of CA1 LFP recordings, depicting IESs (red arrows) before and after CNO i.p. injection in anesthetized fAD mice expressing hM4D(Gi)-mCherry in the nRE-CA1 synapses. Scale bars: 0.5 mV, 20 sec. **g** Violin plot of CA1 IES rate per 1 min bins (*P* <0.0001, Mann-Whitney two-tailed test, same data as in Fig. 2f). **h** Mice were injected with a retrograde viral vector carrying iCre in the CA1 stratum lacunosum moleculare (SLM) and with a control viral vector carrying Cre-dependent mCherry in the nRE, consequently expressing mCherry only in nRE cells that send projections to the CA1. **i** Rate of CA1-IES after VEH (saline) vs CNO i.p. injections (5 mg/kg) in control group of anesthetized fAD mice expressing mCherry in the nRE-CA1 synapses (n = 5 mice, *P* = 0.625, Wilcoxon two-tailed test). ns – non-significant, **P* < 0.05, *****P* < 0.0001. Error bars represent SEM.

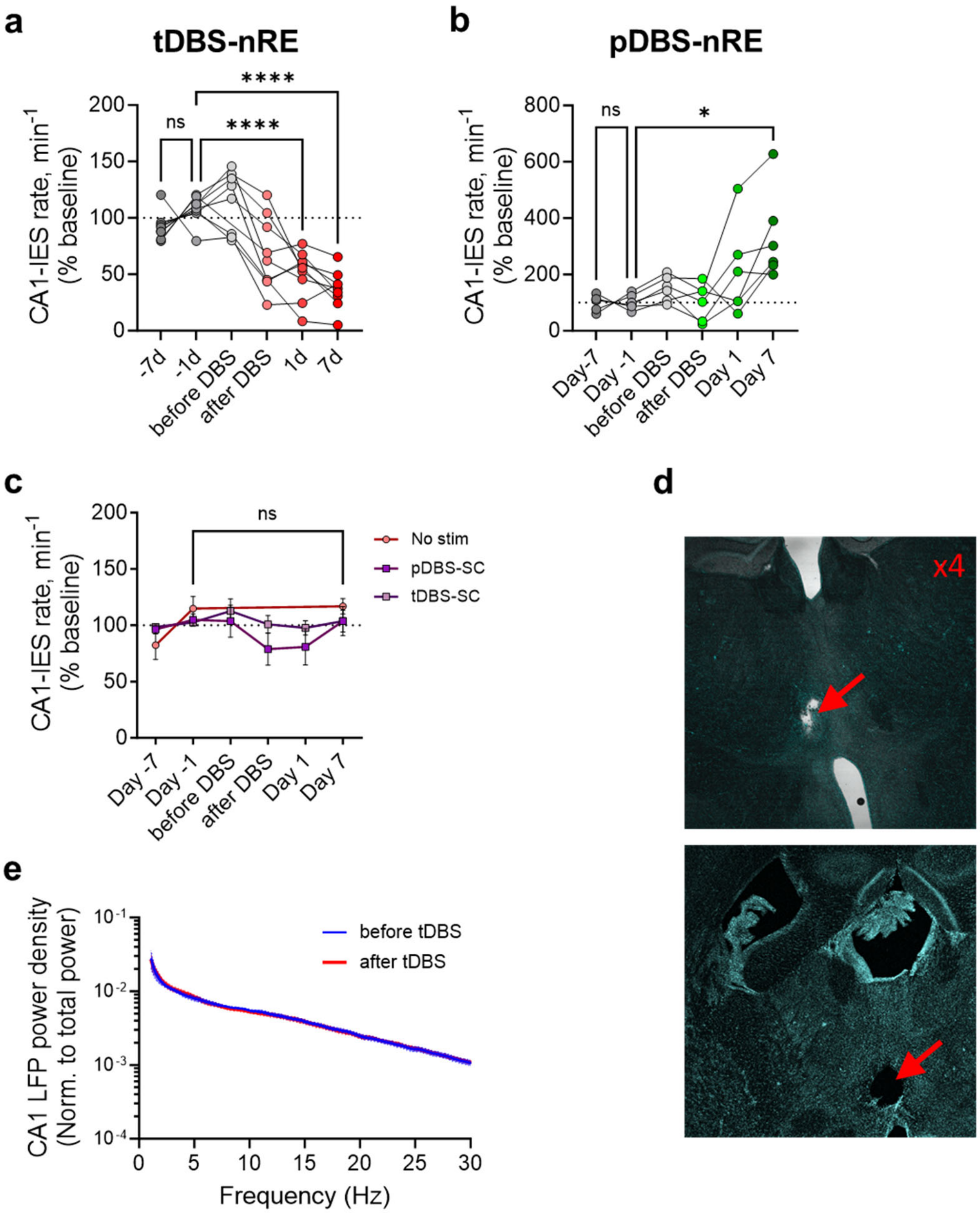

Figure S4 | | Bi-directional regulation of CA1 hyperexcitability by tonic versus phasic nRE stimulation under anesthesia in fAD mice. a fAD mice displayed lower frequency of IES in CA1 during GA following tDBS in the nRE (P <0.0001, mixed effect analysis with Sidak's multiple comparison tests: -1d vs -7d P = 0.4186, -1d vs +1d or +7d P <0.0001, n = 9). **b** fAD mice displayed higher frequency of IES in CA1 during GA following pDBS in the nRE (P = 0.0283, mixed effect analysis with Sidak's multiple comparison tests: -1d vs -7d P = 0.987, -1d vs 7d P = 0.021; n = 6). **c** fAD mice didn't display a significant change in the frequency of IES in CA1 during GA following sham, pDBS or tDBS in the SC. (P = 0.899, mixed effect analysis with Sidak's multiple comparison tests: Day -1 vs. Day 7 P > 0.9 for all groups; n = 6, 4 and 4 for sham, pDBS

and tDBS) **d** Representative histology pictures of fAD mice brains after DBS experiment. Red arrows mark the stimulation electrodes location in the nRE. **e** CA1 LFP power spectra during GA before (blue line) and after (red line) tDBS-nRE in APP/PS1 mice (n = 5). ns – non-significant, **P* < 0.05, *****P* < 0.0001. Error bars represent SEM.

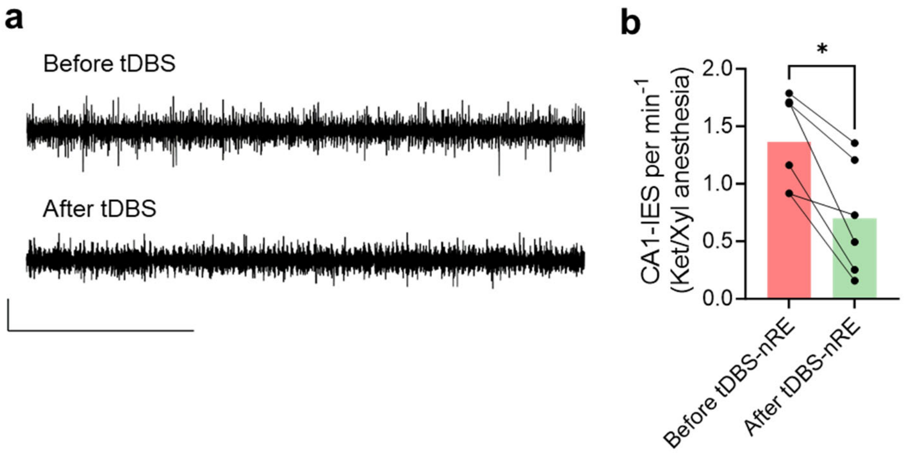

Figure S5 | tDBS-nRE suppresses CA1-IESs under ketamine-xylazine anesthesia. a Representative CA1 LFP recordings during ketamine-xylazine anesthesia before and after tDBS-nRE. Scale bars: 1 mV, 30 sec. **b** Summary data across 6 mice (P = 0.031, Wilcoxon two-tailed test). * $P < 0.05$.

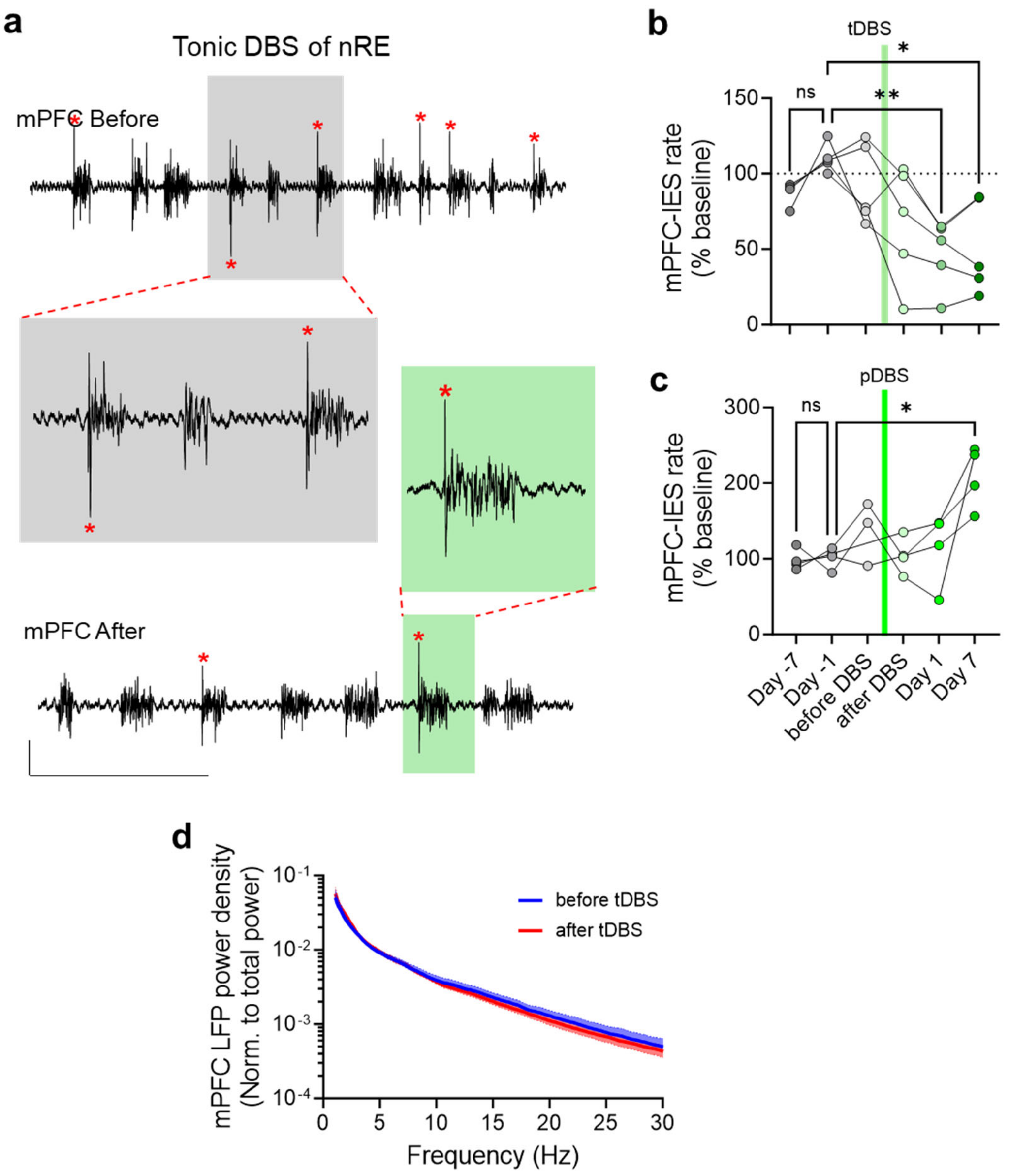

Figure S6 | Bi-directional regulation of mPFC hyperexcitability by tonic versus phasic nRE stimulation under anesthesia in fAD mice. a Representative traces of LFP recordings in the mPFC, depicting IES in the baseline (*top, gray*) and after nRE tonic DBS (*bottom, green*) in anesthetized fAD mice. Scale bars: 20 sec, 0.5 mV. **b** fAD mice displayed lower frequency of IES in the mPFC during GA following tDBS in the nRE (P = 0.0076, mixed effect analysis with Sidak's multiple comparison tests: -1d vs -7d P = 0.171, -1d vs +1d P = 0.009, -1d vs +7d P = 0.046, n = 5). **c** fAD mice displayed higher frequency of IES in the mPFC during GA following tDBS in the nRE (P = 0.028, one way ANOVA with Sidak's multiple comparison tests: -1d vs -7d P = 0.979, -1d vs +7d P = 0.041, n = 4). **d** mPFC LFP power spectra during GA before (blue line) and after (red line) tDBS-nRE in APP/PS1 mice (n = 5). ns – non significant, *P < 0.05, **P < 0.01.

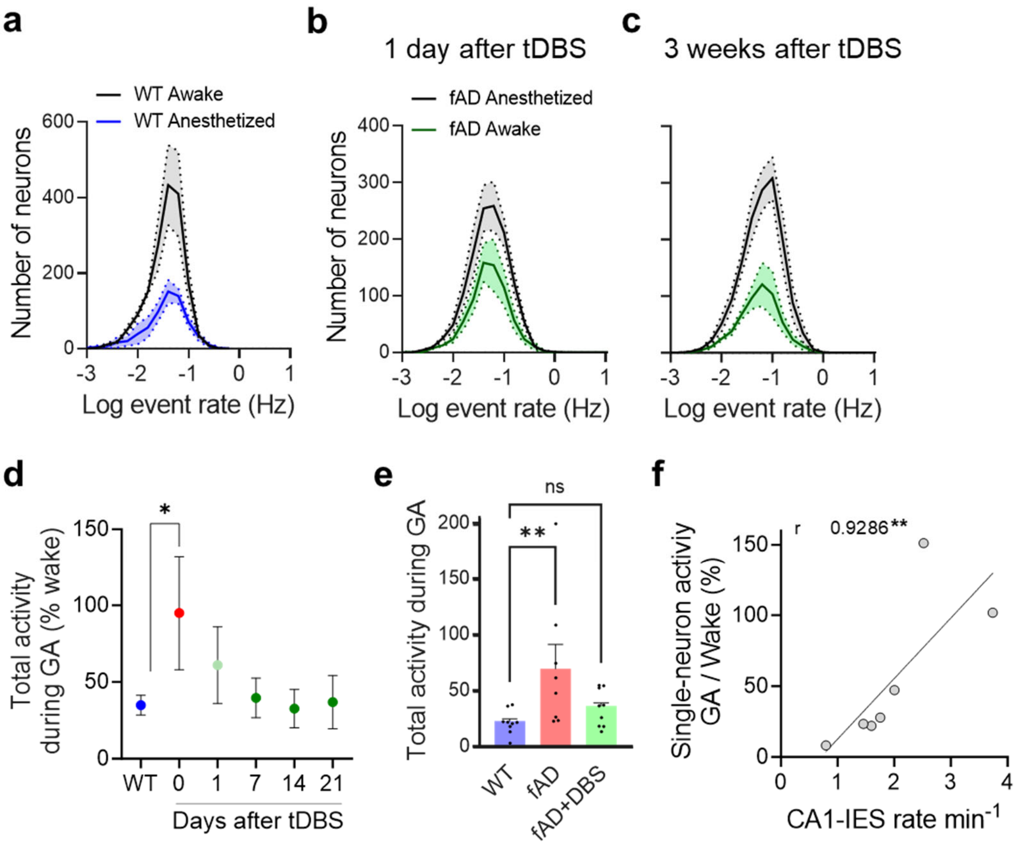

Figure S7 | Tonic nRE stimulation restores anesthesia-induced dyshomeostasis of CA1 activity in fAD mice. a Effect of general anesthesia on average Ca^{2+} event rate distributions of CA1 neuronal populations in WT mice (n = 3). **b-c** Effect of general anesthesia on average Ca^{2+} event rate distributions of CA1 neuronal populations in fAD mice 1 day (b) and 3 weeks (c) after tDBS-nRE. **d** Single-neuron activity (mCaR*Na) during anesthesia, normalized to the activity during awake at the same day, of WT and fAD before and after tDBS-nRE (one-way ANOVA with Sidak's multiple comparison tests: WT vs. fAD -7 days P = 0.0466, WT vs. fAD +7, 14, 21, 28 days P > 0.9; WT n = 9, fAD n = 3). **e** Single-neuron activity (mCaR*Na) during anesthesia was higher in fAD mice compared to WT before DBS, but similar to WT after DBS (P = 0.0014, Kruskel-Wallis test with Dunns' multiple comparison tests: WT vs. fAD P = 0.009, n = 3, WT vs. fAD+DBS P = 0.26, n = 3; dots represent recording sessions). **f** Single-neuron activity (mCaR*Na) during anesthesia, normalized to the activity during awake at the same day, is correlates to the frequency of CA1 IESs during the same session of anesthesia (Spearman r = 0.93, P = 0.0067). ns – nonsignificant, *P < 0.05, **P < 0.01. Error bars and bands represent SEM.

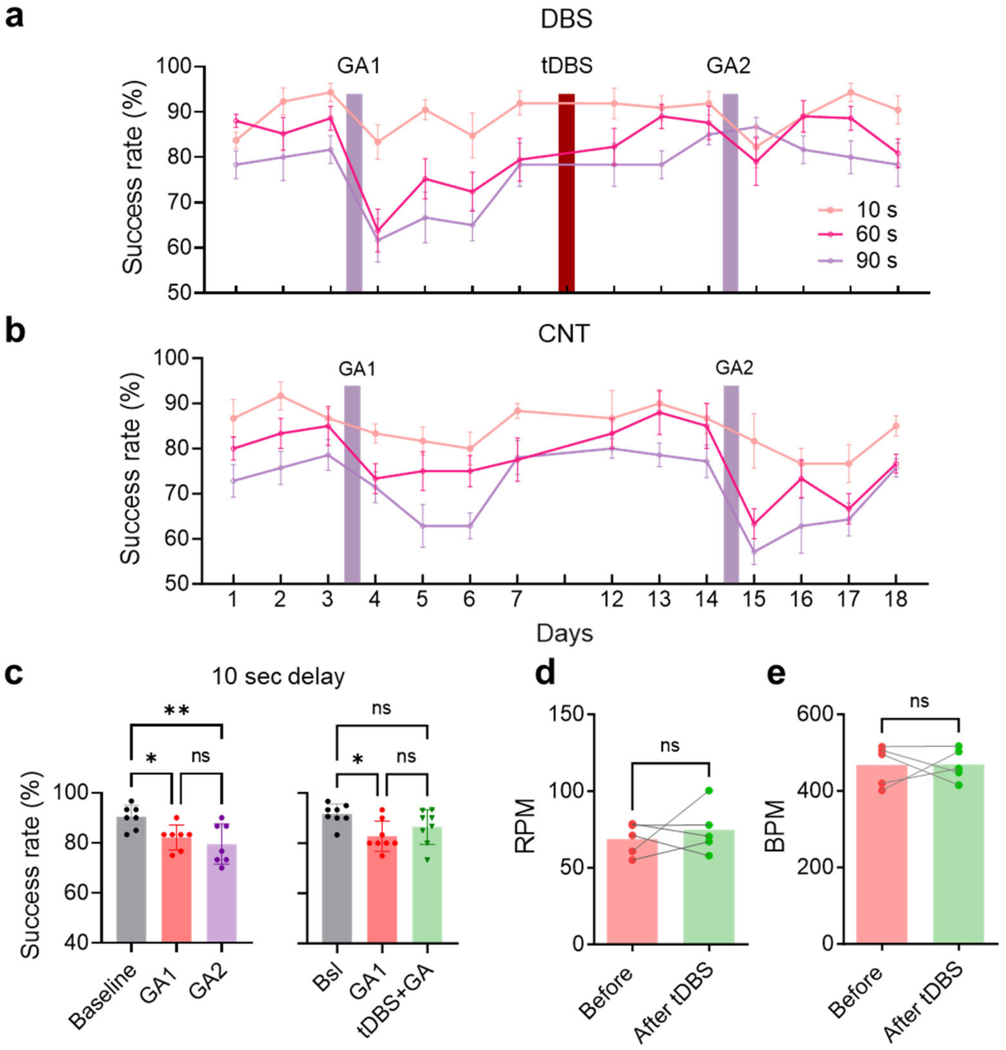

Figure S8 | Tonic nRE stimulation rescues anesthesia-induced impairments of spatial working memory in fAD mice. a Summary data showing the effect of GA on delta maze success rate before and after tonic DBS in fAD mice for 10, 60 (n = 8) and 90 sec (n = 7) delay intervals. **b** Summary data showing the effect of 2 consecutive sessions of GA on delta maze success rate in fAD mice for 10, 60 and 90 sec delay intervals (n = 6). **c** Left: GA-induced impairments of SWM in delta maze, at 10 sec delay, following a second round of anesthesia in the same mice were similar to the first anesthesia (Friedman test with Dann's multiple comparison tests: baseline vs 1st GA: *P* = 0.049, GA1 vs GA2: *P* = 0.004, baseline vs 2nd GA: *P* > 0.9999; n = 6.). Right: GA-induced impairment of SWM in delta maze, at 10 sec delay, was rescued by tDBS-nRE preceding isoflurane exposure (One-way ANOVA with Sidak's multiple comparison tests: baseline vs 1st GA: *P* = 0.049, GA1 vs GA2: *P* = 0.64, baseline vs 2nd GA: *P* = 0.24; n = 8). **d** No difference in mean respiration per minute (RPM) during anesthesia before and after tDBS-nRE (*P* = 0.8125, Wilcoxon two-tailed test, n = 5). **e**, No difference in heart rate, measured by mean beats per minute (BPM),

during anesthesia before and after tonic 25 Hz nRE DBS (*P* >0.9999, Wilcoxon two-tailed test, n = 5). ns - non significant, *P < 0.05, **P < 0.01. Error bars represent SEM.

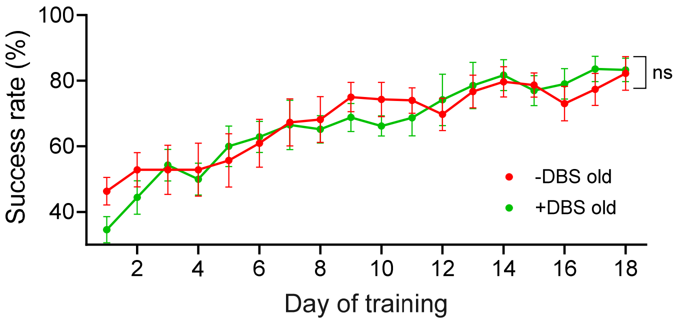

Figure S9 | **Learning curve in 8-9 month old APP/PS1 mice (related to Fig. 7).** Learning curve of SWM during delta-maze training was similar between groups in APP/PS1 mice (*P* = 0.81; Two-way ANOVA, -DBS n = 7, +DBS n = 7).

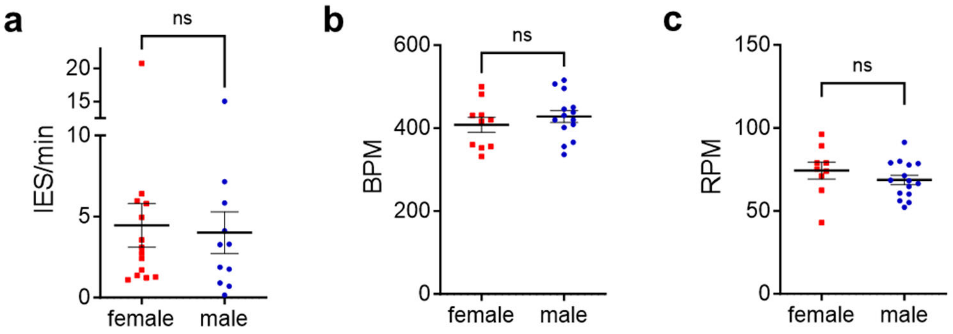

Figure S10 | Electrophysiological and physiological measurements of male and female APP-PS1 mice during anesthesia. a Males and females displayed similar frequency of IESs. (male n = 11, female n = 14. Mann-Whitney two-tailed test. P = 0.8089). **b** No difference in heart rate during anesthesia, measured by mean beats per minute (BPM) between males and females (P = 0.8432; unpaired two-tailed t-test; male n = 14, female n = 10). **c** No difference in mean respiration per minute (RPM) during anesthesia between males and females (P = 0.2682; unpaired two-tailed t-test; male n = 15, female n = 9). Error bars represent SEM.
