# Supplementary material for: Deep brain stimulation of thalamic nucleus reuniens promotes neuronal and cognitive resilience in an Alzheimer’s disease mouse model
Source: Nat Commun. 2023 Nov 2;14:7002. doi: 10.1038/s41467-023-42721-5 (PMC10622498; doi:10.1038/s41467-023-42721-5)
Supplement: Supplementary file 3 — Reporting Summary [file 41467_2023_42721_MOESM3_ESM.pdf]

nature portfolio

Corresponding author(s): Inna Slutsky

Last updated by author(s): 2023.10.15

Reporting Summary

Nature Portfolio wishes to improve the reproducibility of the work that we publish. This form provides structure for consistency and transparency in reporting. For further information on Nature Portfolio policies, see our Editorial Policies and the Editorial Policy Checklist.

Statistics

For all statistical analyses, confirm that the following items are present in the figure legend, table legend, main text, or Methods section.

n/a	Confirmed	
☐	☒	The exact sample size (*n*) for each experimental group/condition, given as a discrete number and unit of measurement
☐	☒	A statement on whether measurements were taken from distinct samples or whether the same sample was measured repeatedly
☐	☒	The statistical test(s) used AND whether they are one- or two-sided<br>*Only common tests should be described solely by name; describe more complex techniques in the Methods section.*
☒	☐	A description of all covariates tested
☐	☒	A description of any assumptions or corrections, such as tests of normality and adjustment for multiple comparisons
☐	☒	A full description of the statistical parameters including central tendency (e.g. means) or other basic estimates (e.g. regression coefficient) AND variation (e.g. standard deviation) or associated estimates of uncertainty (e.g. confidence intervals)
☐	☒	For null hypothesis testing, the test statistic (e.g. *F*, *t*, *r*) with confidence intervals, effect sizes, degrees of freedom and *P* value noted<br>*Give P values as exact values whenever suitable.*
☒	☐	For Bayesian analysis, information on the choice of priors and Markov chain Monte Carlo settings
☐	☒	For hierarchical and complex designs, identification of the appropriate level for tests and full reporting of outcomes
☐	☒	Estimates of effect sizes (e.g. Cohen's *d*, Pearson's *r*), indicating how they were calculated

Our web collection on statistics for biologists contains articles on many of the points above.

Software and code

Policy information about availability of computer code

Data collection	Extracellular field potentials were amplified x100 using Model 1700 by A-M Systems amplifier, band-pass filtered between 0.1 Hz and 5 KHz, and digitized by Digidata 1440A at 56 kHz sampling rate (Molecular Devices).<br>Physiological measurements (BSR, RPR) were collected using 75-1500 physiological monitoring system (Harvard Apparatus).<br>Ca2+ imaging data was collected using nVista 3.0 (Inscopix).<br>cFos imaging was preformed using VERSA8 slide scanner (Leica Aperio).<br>For snRNA-seq 10X Genomics (chemistry V3), Illumina NextSeq 2000.
Data analysis	Physiological measurements: 75-1500 physiological monitoring system (Harvard Apparatus);<br>LFP and fEPSP data: Clampfit 10.7 (Molecular devices) and MATLAB;<br>Ca2+ imaging: Inscopix data processing software (Inscopix) and Matlab - using the constrained non-negative matrix factorization algorithm for endoscopic recordings (CNMF-E) and custom functions (MathWorks);<br>cFos imaging and histology: ImageJ;<br>Statistical analysis: GraphPad Prism 9.<br>Data analysis of the single-nuclei RNA sequencing was done in: CellRanger - version 5.0.0, CellBender; Seurat - a free open-source program - version 4.1.0; DoubletFinder_v3 ; Python's sklearn package; R rstatix package; Harmony package v.0.1.0.

For manuscripts utilizing custom algorithms or software that are central to the research but not yet described in published literature, software must be made available to editors and reviewers. We strongly encourage code deposition in a community repository (e.g. GitHub). See the Nature Portfolio guidelines for submitting code & software for further information.

Data

Policy information about availability of data

All manuscripts must include a data availability statement. This statement should provide the following information, where applicable:

- Accession codes, unique identifiers, or web links for publicly available datasets
- A description of any restrictions on data availability
- For clinical datasets or third party data, please ensure that the statement adheres to our policy

The authors declare that the main data supporting the findings of this study are available in the paper. Datasets generated during the study will be shared by the lead contact upon request. Gene expression dataset is deposited in GEO database under accession code GSE245201.

Human research participants

Policy information about studies involving human research participants and Sex and Gender in Research.

Reporting on sex and gender	*Use the terms sex (biological attribute) and gender (shaped by social and cultural circumstances) carefully in order to avoid confusing both terms. Indicate if findings apply to only one sex or gender; describe whether sex and gender were considered in study design whether sex and/or gender was determined based on self-reporting or assigned and methods used. Provide in the source data disaggregated sex and gender data where this information has been collected, and consent has been obtained for sharing of individual-level data; provide overall numbers in this Reporting Summary. Please state if this information has not been collected. Report sex- and gender-based analyses where performed, justify reasons for lack of sex- and gender-based analysis.*
Population characteristics	*Describe the covariate-relevant population characteristics of the human research participants (e.g. age, genotypic information, past and current diagnosis and treatment categories). If you filled out the behavioural & social sciences study design questions and have nothing to add here, write "See above."*
Recruitment	*Describe how participants were recruited. Outline any potential self-selection bias or other biases that may be present and how these are likely to impact results.*
Ethics oversight	*Identify the organization(s) that approved the study protocol.*

Note that full information on the approval of the study protocol must also be provided in the manuscript.

Field-specific reporting

Please select the one below that is the best fit for your research. If you are not sure, read the appropriate sections before making your selection.

☒ Life sciences ☐ Behavioural & social sciences ☐ Ecological, evolutionary & environmental sciences

For a reference copy of the document with all sections, see nature.com/documents/nr-reporting-summary-flat.pdf

Life sciences study design

All studies must disclose on these points even when the disclosure is negative.

Sample size	The sample size was chosen on the basis of our prior study that showed significant effects with similar sample sizes (Zarhin et al. 2022).
Data exclusions	For all experiments, mice that showed signs of injury, pain or disease were excluded from the experiment. For experiments involving electrodes implantation, animals that postmortem histology revealed a wrong location of electrodes were excluded. Sessions of unstable anesthesia were excluded from the analysis.
Replication	There were 3 replicated experiments in this study:<br>1) Effect of anesthesia on behavior - Fig. 1e and Fig. 6b;<br>2) Effect of tDBS-nRE on eileptiform spikes: Fig. 3h,j and Fig. 6f,g.<br>3) Replication of published results from our previous study (Zarhin et al., 2022) on induction of epileptiform spikes (Fig. 1a-b) and CA1 activity dysregulation (Fig. 5c,g) by general anesthesia in APP/PS1 mice.
Randomization	Samples were chosen randomly from each genotype per treatment per time point.
Blinding	The majority of the experiments were not blinded due to irrelevance or lack of technical feasibility (when perturbations, measurements and analysis in the same animal were done by a single person). For the experiments that were performed by 2 researchers (Fig. 1f and Fig. 7), each of them was blinded to the findings of the other. When possible (Fig. 2, 3j-k, 4u, 6i-k), the researcher was blinded to the timing of perturbations during data analysis.

Reporting for specific materials, systems and methods

We require information from authors about some types of materials, experimental systems and methods used in many studies. Here, indicate whether each material, system or method listed is relevant to your study. If you are not sure if a list item applies to your research, read the appropriate section before selecting a response.

Materials & experimental systems

n/a	Involved in the study
☐	☒ Antibodies
☒	☐ Eukaryotic cell lines
☒	☐ Palaeontology and archaeology
☐	☒ Animals and other organisms
☒	☐ Clinical data
☒	☐ Dual use research of concern

Methods

n/a	Involved in the study
☒	☐ ChIP-seq
☒	☐ Flow cytometry
☒	☐ MRI-based neuroimaging

Antibodies

Antibodies used	c-Fos antibody (rabbit, Synaptic Systems, Cat.No. 226 003; 1:10,000) and secondary antibody (goat anti-rabbit, DyLight 488, Jackson Laboratories; 1:600)
Validation	Commercial c-Fos antibody was validated by the manufacturers and used in references found here: https://sysy.com/product-factsheet/SySy_226003

Animals and other research organisms

Policy information about studies involving animals; ARRIVE guidelines recommended for reporting animal research, and Sex and Gender in Research

Laboratory animals	Experiments were performed on 4-5 and 8-9 month old APP/PS1 (APPSwe/PS1delta9) hemizygotes (Stock No. 005864, The Jackson Laboratory) on a C57BL/6J-congenic background, and their wild-type littermates.
Wild animals	No wild animals were used in this study.
Reporting on sex	Both male and female mice were employed for electrophysiological and Ca2+ imaging measurements. Due to the complexity of the behavioral experiments, combined with electrophysiology, and taking into account the substantial body of prior behavioral studies conducted in our lab and within the broader literature, we utilized male mice for behavioral assessments.
Field-collected samples	No field collected samples were used in this study.
Ethics oversight	All animal experiments were approved by the Tel Aviv University Committee on Animal Care (approvals 01-19-036 and TAU-MD-IL-2204-141-5).

Note that full information on the approval of the study protocol must also be provided in the manuscript.
